# Trastuzumab in early curative breast cancer: A target trial emulation benchmarked against two randomized clinical trials

**DOI:** 10.1371/journal.pmed.1004661

**Published:** 2025-07-21

**Authors:** Vanessa Voelskow, Xabier Garcia-Albeniz, Anita Berglund, Maria Feychting, Tobias Kurth, Anthony A. Matthews

**Affiliations:** 1 Institute of Public Health, Charité—Universitätsmedizin Berlin, Berlin, Germany; 2 Unit of Epidemiology, Institute of Environmental Medicine, Karolinska Institutet, Stockholm, Sweden; 3 RTI Health Solutions, Barcelona, Spain; University of Pennsylvania Perelman School of Medicine, UNITED STATES OF AMERICA

## Abstract

**Background:**

Benchmarking an observational analysis against a randomized trial can increase confidence in the use of observational data to complement inferences made in trials. Until now, few examples of benchmarking have been within oncology. However, benchmarking trials of a cancer treatment poses a unique set of challenges, such as defining composite outcomes like disease-free survival.

**Methods and findings:**

We designed a target trial with a protocol as similar as possible to the B-31 and N9831 randomized trials, which estimated the effect of adjuvant trastuzumab plus chemotherapy compared with chemotherapy alone in individuals with early human epidermal growth factor receptor 2-positive breast cancer. We then carried out an observational analysis by emulating the target trial using routinely collected data from Swedish registries to understand if we can estimate a similar effect of trastuzumab as the trial. The primary endpoint was the composite of disease-free survival consisting of the earliest of (1) local or regional recurrences, (2) distant recurrences, (3) contralateral breast cancer, (4) other second primary cancer, or (5) death from any cause. Individuals who had data compatible with both treatment strategies at baseline were cloned and one copy was assigned to each arm. We applied inverse probability weights to adjust for baseline and time-varying confounding (e.g., age and hematological events like neutropenia). Our observational analysis included 1,578 women, with a median age of 59 years, and who were diagnosed between 2008 and 2015. We estimated a similar effect after five years of follow-up (RR: 0.54, 95% CI [0.44, 0.67]) for the composite endpoint of disease-free survival as the two jointly analyzed B-31 and N9831 trials (HR: 0.48, 95% CI [0.39, 0.59]). While the comparability of results increases confidence in our estimates, there remains a risk of residual and unmeasured confounding, as is the case with all observational analyses.

**Conclusions:**

We successfully benchmarked an observational analysis against the B-31 and N9831 trials. By aligning protocols and using appropriate methodological approaches, we show that observational data can be used to estimate similar results as randomized trials of cancer treatments, like trastuzumab. This opens the door to using observational data to complement results from randomized trials of cancer treatments which can provide quick, cheap, and robust evidence to support decision-making where trials leave evidence gaps.

## Author summary

**Why was this study done?:**

- Observational analyses can complement results from randomized controlled trials by providing evidence on research questions that they could not answer.
- For example, observational data can fill gaps in knowledge for vulnerable people, such as older individuals, but this is often hampered by a lack of trust in these data.
- In addition, oncological studies come with their own set of challenges, such as defining complex outcomes like disease-free survival, aligning a unique time point for treatment initiation in sequences of treatment (e.g., surgery and first-line treatment), and assigning individuals to a treatment when the classification is not distinguishable from the observational data.

**What did the researchers do and find?:**

- We conducted an observational analysis that closely mimicked the design of two jointly analyzed trials (B-31 and N9831) on the effect of trastuzumab in early curative breast cancer and compared results, i.e., benchmarking.
- Our observational analysis included 1,578 women diagnosed between 2008 and 2015.
- We estimated a similar effect after 5 years of follow-up for the composite endpoint of disease-free survival as the two trials. That is, both analyses agreed that trastuzumab substantially increases the 5-year disease-free survival.

**What do these findings mean?:**

- Of course, we cannot rule out the possibility that unmeasured factors, such as frailty, may have influenced our findings, and that the observed similarity is a chance finding.
- However, the comparability of results increases confidence in our estimates, and we also used other indirect methods (e.g., negative control outcome) that could have indicated this possibility, but instead confirmed our findings
- Therefore, the findings show that observational data can be used to estimate similar results as randomized trials of cancer treatments, like trastuzumab.
- This opens the door to using observational data to complement results from randomized trials of cancer treatments which can provide quick, cheap, and robust evidence to support decision-making where trials leave evidence gaps.

## Abstract 3

Vanessa Voelskow and her colleagues emulate a target trial to determine whether an observational analysis could successfully replicate the estimated effects of trastuzumab on overall and disease-free survival.

## Introduction

Benchmarking an observational analysis against a randomized trial increases confidence in the use of observational data to complement inferences made in trials [1]. If one can first replicate the results of a randomized trial using observational data, clinicians and other decision makers may be more confident in the use of these data to address questions not answered by the trials, e.g., the effect of treatment in populations excluded or underrepresented in the trials [2–4]. And, when benchmarking fails and observational data cannot reliably estimate similar results to a trial, we enhance the understanding of the limitations of observational data to answer causal questions.

Until now, few examples of benchmarking have been within oncology [5,6]. However, benchmarking an observational analysis against a trial of a cancer treatment poses a unique set of challenges. First, composite outcomes such as disease-free survival are commonly used, which can be hard to define in observational data. Second, correct alignment of time zero in sequences of treatment (e.g., surgery and first-line treatment) is crucial, as treatment delays may lead to a worse prognosis [7]. Third, intended treatment strategies are not always clearly distinguishable at baseline in observational data, thus the risk of selection bias due to immortal time is high without appropriate study design considerations (i.e., cloning) [7].

Here, we design a target trial [2,4,8,9] with a protocol as similar as possible to the B-31 and N9831 trials [10–12], which estimated the effect of adjuvant trastuzumab plus chemotherapy compared with chemotherapy alone on disease-free and overall survival in individuals with early human epidermal growth factor receptor 2 (HER2)-positive breast cancer. We then emulate the target trial to understand if we can undertake an observational analysis that successfully replicates estimates of the effect of trastuzumab on both overall and disease-free survival using the Swedish National Quality Register for Breast Cancer and the routinely collected data from the Swedish population-based registers.

## Methods

### Index randomized trials: B-31 and N9831

The protocol used for benchmarking is outlined in Table 1. It essentially merges the B‑31 and N9831 trials (S1 Table), as they were similar and analyzed in a joint analysis approved by the Food and Drug Administration and the National Cancer Institute before any data cut-off [10–14]. Both trials were open-label, multicenter, randomized, controlled clinical trials in individuals with early HER2-positive breast cancer who underwent surgery for complete resection of the primary tumor (Table 1). Participants in both studies were randomly assigned to receive either an initial anthracycline-based combination chemotherapy for 12 weeks followed by a taxane-based chemotherapy plus trastuzumab, or the same chemotherapy regimen without trastuzumab. The primary endpoint was the composite of disease-free survival consisting of the earliest of (1) local or regional recurrences, (2) distant recurrences, (3) contralateral breast cancer, (4) other second primary cancer, or (5) death from any cause. Secondary endpoints were the individual components of the composite outcome.

**Table 1:** Summary of protocol used for benchmarking, the target trial and its emulation to compare trastuzumab plus chemotherapy with chemotherapy, NKBC and seven further Swedish registers, 2008–2015.

Protocol component	Protocol of trials B31 and N9831 used for benchmarking, approximately merging components where different	Target trial	Target trial emulation using NKBC and seven other national Swedish registries
Benchmark eligibility criteria	• Adults (≥ 18 years) with confirmed HER2-positive (by IHC or ISH) invasive adenocarcinoma of the breast • Tumor staged as T3M0 or lower (except Tis) by clinical and pathologic evaluation, with either node-positive (up to N1) or high-risk node-negative^a^ disease, defined via either: • N0 and T2 or T3 (irrespective of hormonal status), or • N0 and T1 plus ER- and PR-negative status • Complete resection of the tumor (R0 resection) by either a total mastectomy or a lumpectomy, both combined with an axillary-node dissection requiring clear resection margins • Study period between February 2000 and March 2005, and first endpoint follow-up after six months • Life expectancy at least 10 years except breast cancer diagnosis • Not more than 84 days between surgery and treatment assignment • No bilateral malignancy or anything suspicious of such malignancy • No prior history of breast cancer, including DCIS, except LCIS • History of non-breast malignancies is no reason for exclusion if disease-free for at least 5 years prior to baseline, or if diagnosed and treated within the past 5 years for one of the following cancers: carcinoma in situ of the cervix, melanoma in situ, and basal cell and squamous cell carcinoma in situ of the skin • No concurrent treatment with other investigational agent (i.e., pertuzumab) • No contraindication to any of the study drugs, as follows: ***Active cardiac diseases*** • Angina pectoris that requires the use of antianginal medication, cardiac arrhythmia requiring medication, or current use of digitalis or beta-blockers for congestive heart failure • Clinically significant pericardial effusion, cardiomegaly on chest X-ray, severe conduction abnormality, clinically significant valvular disease, ventricular hypertrophy on EKG, or LVEF outside the normal range • Poorly controlled hypertension ***History of cardiac diseases*** • Myocardial infarction, congestive heart failure, or cardiomyopathy ***Non-cardiac contraindications*** • Hematopoietic, renal, or hepatic disorders based on laboratory values	*Same as merged eligibility criteria of trials B31 and N9831 used for benchmarking apart from:* • Tumor staging based on clinical examinations only • Study period between January 2008 and December 2015, and potential for at least half a year of follow-up • Any life expectancy • No prior history of any breast cancer (including DCIS and LCIS) • No diagnosis of any non-breast malignancy in last 5 years except carcinoma in situ of the cervix, melanoma in situ, and non-melanoma skin cancers staged as no more than TisN0M0 or staged as in situ according to SNOMED or FIGO • No concurrent treatment with pertuzumab or other HER2 targeted monoclonal antibodies • No assessment of clinical significance or severity, chest X-rays, LVEF, hypertension, or renal and hepatic values (e.g., excluded individuals with any record suggestive of cardiomegaly or pericardial effusion)	*Same as target trial* • All eligibility criteria identified in the NKBC unless otherwise stated in S2 Table. • To capture contraindications, active disease was operationalized as any record suggestive of this contraindication within 90 days before baseline, and we had a look back in the registers to identify individuals with a history of cardiac diseases of 10 years
Treatment strategies^b^	(1) Initiation of trastuzumab concomitant to a taxane-based chemotherapy after 12 weeks of receiving anthracycline-based combination chemotherapy (2) No initiation of trastuzumab over follow-up while receiving the same chemotherapy regime as arm (1) Chemotherapies administered, doses, and durations of treatment: • Anthracycline-based combination chemotherapy: doxorubicin (60 mg/m2) and cyclophosphamide (600 mg/m2) every 21 days for four cycles • Taxane based chemotherapy: paclitaxel à 175 mg/m^2^ every 3 weeks for four cycles or at 80 mg/m2 for 12 weekly doses at the investigator’s discretion • Trastuzumab: loading dose of 4 mg/kg, followed by weekly doses of 2 mg/kg for 51 weeks	(1) Initiation of trastuzumab within 12 weeks (or within a 2-week grace period at the latest) after start of receiving chemotherapy (2) No initiation of trastuzumab over follow-up while receiving the same chemotherapy regime as in the intervention arm No restriction on types or order of chemotherapies (e.g., anthracyclines and taxanes), number of cycles, doses, and durations of treatment; radiation and hormonal therapy can be received at the discretion of the treating physician	Same as target trial.
Treatment assignment	Participants are assigned at random to one of the above-described treatment arms and are aware of the assigned strategy	Each individual is assigned at random to one of the two above outlined treatment strategies at baseline and is aware of the assigned strategy	Randomization was assumed conditional on baseline covariates: age, marital status, family income, menopausal status, year of diagnosis, size of tumor at diagnosis, lymph node involvement, estrogen receptor status, progesterone receptor status, size of the largest tumor at surgery, histological grade, number of tumors in breast, and number of visits to healthcare professional within 5 years prior to baseline
Primary and main secondary endpoint	Disease-free survival and overall survival Disease-free survival operationalized as time from randomization until the earliest of (1) local or regional recurrences, (2) distant recurrences, (3) contralateral breast cancer, (4) other second primary cancer, or (5) death from any cause	Same as endpoints of trials B31 and N9831	Same as target trial
Benchmark follow-up	5 years	Same as follow-up of trials B31 and N9831 used for benchmarking	Same as target trial
Causal contrast	Intention-to-treat effect, per-protocol effect	Same as causal contrast of trials B31 and N9831	Observational analog of per-protocol effect
Benchmark statistical analysis	Intention-to-treat analysis and per-protocol analysis, primarily using • Kaplan–Meier method, and • Stratified log-rank test to calculate HRs and depict survival curves after 5 years of follow-up.	Intention-to-treat analysis: Survival in each arm estimated using pooled logistic regression model with adjustment for unbalanced baseline covariates via IP weighting if necessary, with additional IP weighting to adjust for potential selection bias due to loss to follow-up. Risks estimated by one minus survival and then used to plot cumulative incidence curves and contrasted to estimate RD and RRs. Per-protocol analysis: Same, except censored when they deviate from assigned treatment, and IP weighting to adjust for baseline and time-varying variables	Same as target trial for per protocol analysis apart from: • Eligible individuals with data compatible with both treatment strategies cloned at baseline, and each clone assigned to one strategy and followed until they did not adhere. IP weighting to adjust for potential selection bias due to informative censoring. • No additional adjustment for loss to follow-up, as according to the Total Population Register only five individuals migrated out of Sweden

^a^Definition taken from trial N9831; individuals with node-negative disease were excluded in trial B-31.

^b^A third treatment arm in trial N9831 was to evaluate whether trastuzumab be added sequentially or concurrently to a taxane-based chemotherapy. As this question is outside the scope of this article, it is left out in this table to enhance readability. Furthermore, both trials had protocol specifications on radiation therapy and hormonal treatments to be administered, if indicated.

Abbreviations: DCIS: ductal carcinoma in situ; DFS: disease-free survival; EKG: electrocardiogram; ER: estrogen receptor; FIGO: International Federation of Gynecology and Obstetrics cancer staging system; HER: human epidermal growth factor receptor; HR: Hazard Ratio; ICD-10: International Classification of Diseases, Tenth Revision; IHC: immunohistochemistry; IP: inverse probability; ISH: fluorescent in situ hybridization; LCIS: lobular carcinoma in situ; LVEF: left ventricular ejection fraction; NKBC: Swedish National Quality Register for Breast Cancer; PR: progesterone receptor; RD: risk difference; RR: risk ratio; SNOMED: Systematized Nomenclature of Medicine Clinical Terms

After approximately five years in each study (B-31: February 2000 to February 2005; N9831: May 2000 to March 2005), an intention-to-treat analysis was performed [12]. A total of 3,676 individuals were randomized, of whom 3,351 had at least one follow-up examination. 1,672 individuals were assigned to the trastuzumab plus chemotherapy arm (B-31: 864; N9831: 808) and 1,679 to the chemotherapy only arm (B-31: 872; N9831: 807). The 5-year disease-free survival proportion was 92.0% (1539/1672) in the trastuzumab group and 84.5% (1418/1679) in the no trastuzumab group (hazard ratio [HR]: 0.48, 95% confidence interval [CI] [0.39, 0.59]). Overall survival proportion at 5 years were 96.3% (1610/1672) and 94.5% (1587/1679), respectively (HR: 0.67, 95% CI [0.48, 0.93]).

## The observational analysis

### Target trial protocol

To compare the index trial with an observational analysis that attempts to answer the same clinical question, we first specified the protocol of a target trial, with deviations from the index trial protocol only when the observational data did not correspond to the information collected in the trial (Table 1). We outline the main differences herein.

The target trial would not exclude individuals with a life expectancy of less than 10 years, and relevant eligibility criteria would be identified using prior clinical events and drug use rather than biomarker levels (e.g., left ventricular ejection fraction [LVEF]). The treatment strategies would be: (1) initiation of trastuzumab within 12 weeks (plus a 2-week grace period to consider delays in treatment) after receiving chemotherapy, and (2) no initiation of trastuzumab over follow-up while receiving the same chemotherapy regime as in the intervention arm. The type or substances of chemotherapy would not be restricted. Based on previous studies which found interchangeable effects [15–17], we would also not restrict the order of chemotherapy administration (e.g., anthracyclines or taxanes first). The outcomes would be the same as in the B-31 and N9831 trials, and everyone would be followed from the time of assignment (i.e., day 1 of first chemotherapy received) until the earliest of the outcome of interest, loss to follow-up, or five years. Since only 1% of individuals declined chemotherapy after randomization in both the B-31 and N9831trial [12], the time zero in our target trial would be the same as that in the trials. The causal contrasts would be the intention-to-treat and per-protocol effect.

The 5-year survival in each arm for each endpoint would be estimated parametrically using a pooled logistic regression model with an indicator for assigned strategy, time (as linear and quadratic terms), and a product term between the assignment indicator and time. Prognostic factors unbalanced between groups would be used to estimate inverse probability weights if necessary (truncated at their 99th percentile to prevent an influence of outliers). The intention-to-treat analysis would estimate the effect of being assigned to trastuzumab plus chemotherapy versus chemotherapy on the outcome of interest. The per-protocol effect would estimate the effect of receiving and adhering to the assigned strategy of either trastuzumab plus chemotherapy or chemotherapy on the outcomes of interest. To estimate the per-protocol effect, individuals would be censored when they deviate from their assigned treatment strategy, and inverse probability weighting would be used to adjust for baseline and time-varying prognostic factors associated with non-adherence. For both analyses, risks would then be estimated using one minus survival and contrasted to calculate risk differences (RDs) and risk ratios (RRs). Ideally, we would compare the RDs and RRs at five years from the observational analyses with those from the trials. However, the main effect estimate in the trials was the HR, which can greatly vary between studies due to differences in the length of follow-up and the distribution of censoring [18]. Therefore, instead of comparing the effect measures quantitatively, the focus would be on qualitatively evaluating the direction of effects and overall conclusions to decide whether benchmarking was successful.

### The observational emulation of the target trial

We used data from the Swedish National Quality Registry for Breast Cancer to emulate the target trial. The Breast Cancer registry was launched in 2008 to facilitate nationwide collection of data for all primary invasive and in situ breast cancer cases across Sweden [19]. It comprises data on pre-defined diagnostics, therapeutic procedures, and death during follow-up. Individual healthcare providers have the responsibility of reporting to the Registry. Further monitoring of these data is performed by the six Regional Cancer Centers in Sweden [20]. To obtain information on disease recurrence, comorbid conditions, drug use, social, and emigration status, we linked the Breast Cancer Quality Registry to seven nationwide registers for which reporting is regulated by Swedish laws: the National Cancer Register, the National Patient Register (i.e., inpatient and outpatient), the National Cause of Death Register, the National Prescribed Drug Register; the Longitudinal Integration Database for Health Insurance and Labor Market Studies (LISA), and the Total Population Register [21–29]. The analyses are thus based on a total of linked eight registers.

Each component of the target trial protocol was emulated as closely as possible using the above data (see Table 1 for details). Active contraindications were operationalized as any code suggestive of the contraindication within 90 days before baseline. Outcomes were defined as follows (see also S3 Table): local recurrence of breast cancer was identified using surgical procedure codes (rather than a code for diagnosis of local or regional recurrence) from the Inpatient Register; distant recurrences using codes for secondary cancers from the Inpatient and Outpatient Register; contralateral breast cancer from the Breast Cancer Quality Registry; other second primary cancers using codes for malignancies except breast cancer and secondary cancers in the National Cancer Register; and death from the Cause of Death Register. Chemotherapy treatment was identified through the Breast Cancer Quality Registry, in which information was available on whether any type of chemotherapy was administered and if it contained anthracyclines. The start date of chemotherapy was captured only once, when the first substance type was initiated. We, therefore, made the assumption that trastuzumab was only administered concomitant to taxanes, which was based on expert opinion regarding normal practice in Sweden. There were missing data for start date of chemotherapy for 20% of individuals. Imputation was used to impute the time from primary surgery to this date using a Bayesian linear regression model with age, number of additional surgeries, and death as predictors. Data on start date of trastuzumab was complete among individuals who initiated trastuzumab.

As each treatment strategy was not distinguishable at baseline in the observational data, the 3-step clone, censor, and weighting procedure was used [30,31]. The first step was to clone each individual if their data were compatible with both treatment strategies at baseline. Then, when individuals deviated from their assigned strategy, they were censored. That is, in the trastuzumab plus chemotherapy arm individuals were censored at week 14 (i.e., week 12 plus 2 weeks grace period) if they had not initiated trastuzumab; in the chemotherapy only arm individuals were censored at the week they initiated trastuzumab. Time-varying inverse probability weights were then used to account for potential baseline and time-varying confounding and selection bias due to informative censoring. Further details on our modeling approach are in S1 Data. The baseline variables were: age, marital status, family income, menopausal status, year of diagnosis, size of tumor at diagnosis, lymph node involvement, estrogen receptor status, progesterone receptor status, size of the largest tumor at surgery, histological grade, number of tumors in breast, and number of visits to healthcare professional within 5 years prior to baseline. The time-varying variables were: drug intake against cardiac disease, mild to moderate cardiac disease, severe cardiac disease, liver function disorder, renal function disorder, dyspnea, hematological events (e.g., neutropenia), severe infections, neurologic disorders, and gastrointestinal disorders. Time-varying variables were modeled using the week of occurrence and the subsequent week. S4 Table shows full variable definitions and operationalizations. S1 Appendix shows the coefficients from the pooled logistic regression model for the denominator of the inverse probability of treatment weights. Nonparametric bootstrapping with 500 samples was used to calculate 95% confidence intervals.

### Sensitivity analyses

We performed three sensitivity analyses. First, we restricted analyses to individuals who initiated anthracyclines at baseline and who received trastuzumab between weeks 8 and 16. Second, we only included individuals with a known date for start of chemotherapy (later referred to as complete case analysis). Third, we estimated the effect on survival specifically from breast cancer to understand if non-breast cancer deaths led to a distortion of results. All analyses were conducted in R version 4.3.1.

This study was approved by the Regional Ethical Review Board in Stockholm (2011/634-31, 2019-05224). As the data were register-based and pseudonymized, the Ethical Review Board waived the requirement for participant consent. The reporting follows the Strengthening the Reporting of Observational Studies in Epidemiology (STROBE) guideline [32] (S1 Checklist).

## Results

**Fig 1:**
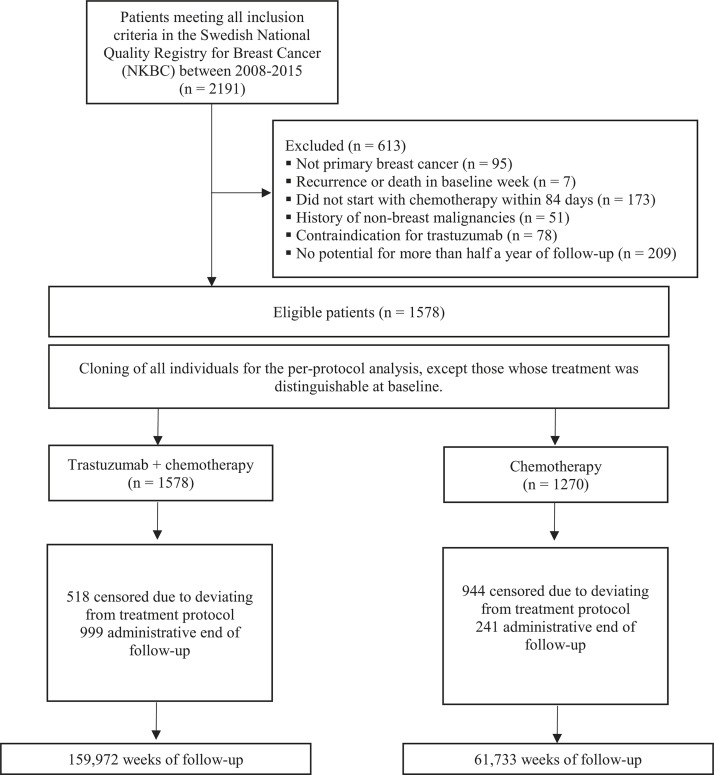
Flowchart of patient selection into the emulation of a target trial comparing trastuzumab plus chemotherapy with chemotherapy, NKBC, and seven further Swedish registers, 2008-2015.

Fig 1 depicts a flowchart of patient selection for the target trial emulation. The baseline characteristics of all 1,578 eligible individuals are shown in Table 2. All but 308 individuals were assigned to both strategies at baseline (i.e., they were only given chemotherapy, and it was not clear if they would go on to be given trastuzumab). Compared with those in the B-31 and N9831 trials, individuals in the target trial emulation were older, but slightly fewer had a tumor larger than 5 cm in diameter. For the overall survival analysis, there were 159,972 and 61,733 person-weeks of follow-up in the trastuzumab plus chemotherapy and chemotherapy arm, respectively.

**Table 2:** Baseline characteristics of individuals included in the emulation of a target trial comparing trastuzumab plus chemotherapy with chemotherapy, NKBC, and seven further Swedish registers, 2008–2015.

	Total (*N* = 1,578)
**Female sex, *n* (%)**	1,578 (100%)
**Age, median (IQR)**	59.0 (48.0, 68.0)
**Age categories in years, *n* (%)**	
≤39	134 (8.5%)
40–49	311 (19.7%)
50–59	381 (24.1%)
≥60	414 (26.2%)
≥70	338 (21.4%)
**Marital status, *n* (%)**	
Not married	308 (19.5%)
Married or registered partner	832 (52.7%)
Divorced or divorced partner	271 (17.2%)
Widow(er) or surviving partner	159 (10.1%)
Missing	8 (0.5%)
**Family income, *n* (%)**	
Individuals with income in the lowest quartile	387 (24.5%)
Individuals with income in the interquartile range	816 (51.7%)
Individuals with income in the highest quartile	367 (23.3%)
Missing	8 (0.5%)
**Result from HER2 immunohistochemistry (IHC) at surgery, *n* (%)**	
0	0 (0%)
1+	0 (0%)
0–1+	3 (0.2%)
2+	25 (1.6%)
3+	96 (6.1%)
Missing or not assessable	1,454 (92.1%)
**Result from HER2 in situ hybridization (ISH) analysis at surgery, *n* (%)**	
Amplified	1,568 (99.4%)
Not amplified	2 (0.1%)
Missing or not assessable	8 (0.5%)
**Side of breast cancer, *n* (%)**	
Right	745 (47.2%)
Left	833 (52.8%)
**Tumor size at diagnosis**^a^ **in cm, *n* (%)**	
≤2.0	427 (27.1%)
>2.0–5.0	1,044 (66.2%)
≥5.1	104 (6.6%)
Missing	3 (0.2%)
**Lymph node involvement at diagnosis** ^a^ **, *n* (%)**	
N0 No regional lymph node metastases	1,184 (75.0%)
N1 Free lymph node metastases in ipsilateral axilla	394 (25.0%)
**ER status, *n* (%)**	
Positive	811 (51.4%)
Negative	762 (48.3%)
Missing	5 (0.3%)
**PR status, *n* (%)**	
Positive	554 (35.1%)
Negative	1,016 (64.4%)
Missing	8 (0.5%)
**Type of surgery, *n* (%)**	
Partial mastectomy	613 (38.8%)
Mastectomy	961 (60.9%)
Subcutaneous mastectomy	4 (0.3%)
**Radiotherapy after surgery, *n* (%)**	
No	2 (0.1%)
Yes	1,576 (99.9%)
**Year of diagnosis, *n* (%)**	
2008	83 (5.3%)
2009	117 (7.4%)
2010	184 (11.7%)
2011	269 (17.0%)
2012	244 (15.5%)
2013	284 (18.0%)
2014	306 (19.4%)
2015	91 (5.8%)

^a^According to TNM staging.

Abbreviations: IQR: interquartile range; *N*: total number of individuals in study population; *n*: number of individuals with characteristic; NKBC: Swedish National Quality Register for Breast Cancer.

**Fig 2:**
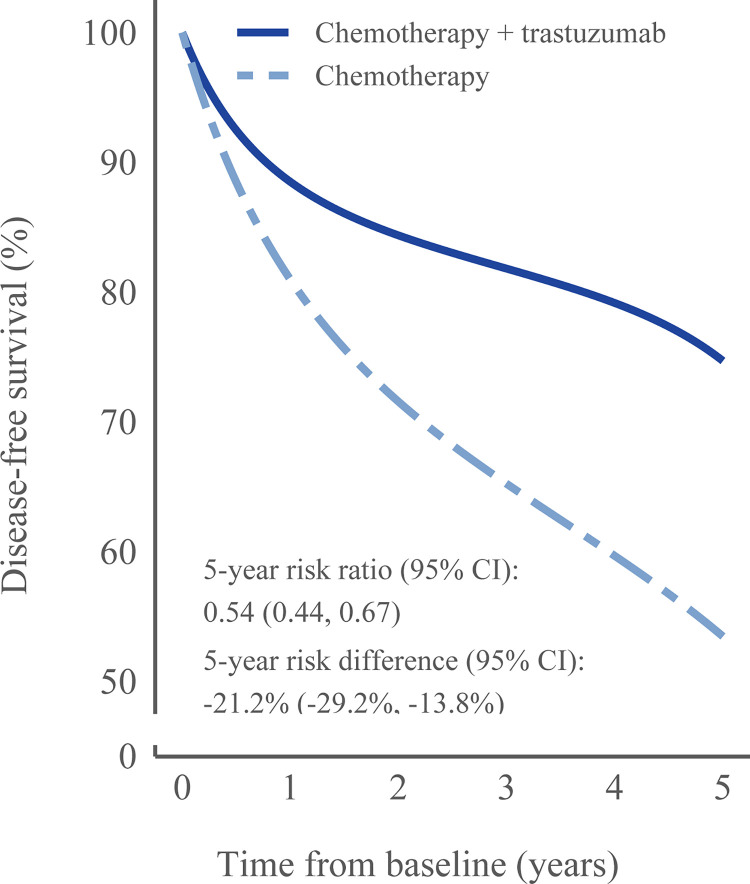
Inverse-probability weighted survival curve of disease-free survival to compare adjuvant chemotherapy + trastuzumab with chemotherapy from an observational emulation of a target trial, NKBC, and seven further Swedish registers, 2008–2015 (main analysis based on 1,578 individuals).

Table 3 shows the estimated survival, RDs, and RRs adjusted for baseline and time-varying variables, and Figs 2, S1, and S2 show the inverse probability weighted survival curves for all outcomes. For the primary analysis 74.7% (95% CI [70.7%, 78.5%]) were disease-free and alive at year 5 in the trastuzumab plus chemotherapy group and 53.5% (95% CI [46.6%, 60.3%]) in the chemotherapy group, resulting in an RD of −21.2% (95% CI [−29.2%, −13.8%]) and an RR of 0.54 (95% CI [0.44, 0.67]). S5 Table shows the distribution of final inverse probability weights.

**Table 3:** Survival, risk differences, and risk ratios at 5 years from baseline estimated in the observational emulation of a target trial comparing trastuzumab plus chemotherapy with chemotherapy, NKBC, and seven further Swedish registers, 2008–2015 (main analysis based on 1578 individuals).

Endpoint	Trastuzumab + chemotherapy		Chemotherapy		Risk Difference, % (95% CI)	Risk Ratio (95% CI)
	Number of events (unique^a^)	Survival, % (95% CI)	Number of events (unique^a^)	Survival, % (95% CI)		
Disease-free survival	211 (134)	74.7 (70.7, 78.5)	177 (100)	53.5 (46.6, 60.3)	−21.2 (−29.2, −13.8)	0.54 (0.44, 0.67)
Overall survival	61 (52)	90.7 (88.1, 93.2)	85 (76)	67.5 (60.1, 74.0)	−23.2 (−30.8, −16.0)	0.29 (0.20, 0.40)
Local recurrence	0	–^b^	5 (5)	98.2 (96.4, 99.5)	–^b^	–^b^
Distant recurrence	169 (103)	80.7 (76.9, 84.1)	115 (49)	73.8 (68.2, 79.0)	−6.9 (−13.6, −0.9)	0.74 (0.56, 0.96)
Contralateral breast cancer	10 (9)	97.9 (96.0, 99.2)	4 (3)	98.6 (96.9, 99.9)	0.8 (−1.4, 3.0)	1.56 (0.44, 17.23)
Other second primary cancer	14 (13)	98.1 (96.8, 99.1)	12 (11)	95.2 (92.1, 97.8)	−2.9 (−6.2, 0.0)	0.39 (0.16, 1.00)

^a^Non-unique events resulting from months in which individuals contributed to both strategies and therefore counted towards both strategies.

^b^Cannot be estimated, as there were no events in the trastuzumab + chemotherapy arm.

Abbreviation: CI: confidence interval.

Results for the individual components of disease-free survival were as follows. Overall survival at 5 years was 90.7% (95% CI [88.1%, 93.2%) in the trastuzumab plus chemotherapy group and 67.5% (95% CI [60.1%, 74.0%]) in the chemotherapy group, resulting in an RD of −23.2% (95% CI [−30.8%, −16.0%]) and an RR of 0.29 (95% CI [0.20, 0.40]). The 5-year distant recurrence free survival was 80.7% (95% CI [76.9%, 84.1%]) in the trastuzumab plus chemotherapy group and 73.8% (95% CI [68.2%, 79.0%]) in the chemotherapy group, resulting in an RD of −6.9% (95% CI [−13.6%, −0.9%]) and an RR of 0.74 (95% CI [0.56, 0.96]). There were few events for the remaining components of disease-free survival (local recurrence, contralateral breast cancer, other second primary cancer). All results from sensitivity analyses were similar to the main analysis (see S6, S7, and S8 Tables, and S3, S4, S5, S6, S7, and S8 Figs).

## Discussion

We used routinely collected data from Swedish population-based registers to emulate a target trial that asked a similar clinical question to the B-31 and N9831 randomized trials. Both the trials and observational analysis estimated similar effects of trastuzumab on disease-free survival and overall survival in women with HER2-positive breast cancer, i.e., a clear increase in survival. Data from both the trials and observational analyses, therefore, support the recommendation of trastuzumab.

Symmetry of conclusions between our observational analysis and the index trials, i.e., successful benchmarking, increases confidence in our observational analysis. However, this does not mean there are no differences between the studies. In fact, several differences affecting estimates in different directions may average out creating a false impression of successful benchmarking. Therefore, we will consider important differences and their potential impact.

A central challenge was to define the individual cancer-specific components of the disease-free survival outcome in the available routinely collected data using definitions similar to the trials. Consider the following two examples: (1) we defined distant recurrence using International Classification of Diseases, Tenth Revision, Tenth Revision (ICD-10) codes that capture secondary cancers, which, in fact, could be a local, regional or distant recurrence; and (2) we defined local recurrences using surgical procedure codes, meaning a local recurrence that was not removed is missed. As there are several unavoidable misclassification problems with these definitions of local and distant recurrences, the survival estimates for trastuzumab of these specific outcomes are difficult to interpret. However, for the composite outcome of disease-free survival, the effect of this misclassification is less problematic. This is because misclassification occurs between the individual components (i.e., capturing local recurrence instead of distant recurrence) or a missing diagnosis for one component could be captured in another component (i.e., missing local recurrences can be captured within the distant recurrence definition). However, the absolute risks for disease-free survival may be lower if any diagnoses of the cancer-specific components are not recorded in the registers.

A differential distribution of treatment effect modifiers between the trial and observational analysis could also be a reason for different estimated effects. In our observational analysis, individuals were, on average, older than those in the trials, and the proportion of individuals with lymph node involvement was not comparable between studies. However, it is unlikely that this contributes to a meaningful difference between effect estimates for disease-free survival as it has been suggested that the effect of trastuzumab on recurrence risk does not differ by age or nodal status [33]. Notwithstanding, an older age is often accompanied by further comorbidities, which we might not have captured in our analyses, and could lead to a poorer response to treatment and/or more treatment-unrelated deaths than in the randomized trials, explaining the more pronounced absolute risk estimates for overall survival.

As with all observational analyses, there is a risk of residual and unmeasured confounding. Our analysis required accounting for both baseline reasons individuals would or would not receive trastuzumab, and post-baseline factors associated with adhering to the assigned treatment strategy (i.e., whether or not an individual initiated trastuzumab over follow-up). We relied on diagnoses of clinical events and drug use to account for this potential confounding. Ideally, we would also use information on biomarkers (e.g., laboratory or radiology parameters), as changes in these recordings could be a reason someone would not receive trastuzumab, but such test results are only routinely captured in the register data for selected conditions. Also, we would need information on treatment discontinuation and dose modifications, which is not captured in the registers. However, if we consider the null result for other second primary cancers as a negative control to assess the impact of potential unmeasured factors (i.e., confounding), there is enhanced certainty that cancer-specific outcomes are not affected.

One notable result from our observational analysis is the large estimated effect of trastuzumab on overall survival, which is considerably larger than the effect estimated in the trials. This difference could be a consequence of unmeasured confounding in the observational analysis, i.e., there were factors associated with overall survival (but not particularly associated with disease-free survival) not captured in the data, such as frailty, that were also reasons someone was withheld trastuzumab. However, there were very few non-breast cancer-related deaths, and the estimated effect on breast cancer-specific survival is similar to the effect on overall survival. So, it is likely that the effect estimates for overall survival reflect a difference in deaths due to the breast cancer.

Another factor contributing to the dissimilarity in the effect estimate for overall survival may be the estimation of the analog of the per-protocol effect in the observational analysis and the estimation of the intention-to-treat effect in the trials. If individuals assigned to the chemotherapy arm in the trials switched to additionally receive trastuzumab, this could lead to an attenuation of the effect estimate on overall survival in an intention-to-treat analysis. It is then plausible that the effect estimate derived from a per-protocol analysis would be larger than that obtained from an intention-to-treat analysis. As the trials did not conduct a per-protocol analysis, the comparison of effect estimates from the observational analysis and the trials should focus more on the direction and the definitiveness of the superiority of trastuzumab plus chemotherapy irrespective of the data source.

A limitation of the observational analyses was that there was 20% missing data for the start date of chemotherapy. To overcome this, we imputed the start date for those with missing data. Although such imputation relies on the untestable missing at random assumption, we also carried out a sensitivity analysis in which we excluded all individuals with missing data, which returned similar results. The consistency between these two approaches to deal with missing data enhances the certainty in our results.

Of note, the person-weeks of follow-up was higher in the trastuzumab plus chemotherapy arm compared with the chemotherapy arm. This is a consequence of more individuals receiving trastuzumab plus chemotherapy than chemotherapy alone (i.e., at week 15, there were 1,050 individuals in the trastuzumab plus chemotherapy arm and 518 in the chemotherapy only arm) and the survival being considerably higher in the trastuzumab group. But this is not expected to bias effect estimates, particularly because we adjust for time-varying factors related to censoring.

If investigators using routinely collected data can successfully benchmark analyses that estimate the effect of cancer treatments on both overall and disease-free survival against a randomized trial, it would increase confidence in the ability of these data to ask questions that could not be answered in the initial trial. For example, routinely collected data can provide evidence more relevant to everyday clinical practice situations. Individuals who are regularly underrepresented or excluded from randomized trials often represent a majority of affected individuals [34,35]; routinely collected data, therefore, provide a unique opportunity to explore heterogeneity in treatment effectiveness across subgroups.

We successfully benchmarked an observational analysis of trastuzumab on disease-free and overall survival against the B-31 and N9831 trials. By aligning protocols and using appropriate methodological approaches, we show that observational data can be used to estimate similar results as randomized trials of cancer treatments like trastuzumab. This opens the door to using observational data to complement results from randomized trials of cancer treatments which can provide quick, cheap, and robust evidence to support decision-making where trials leave evidence gaps.

## Supporting information

S1 Data (DOCX)

S1 Table Summary of selected protocol components of the RCTs B-31 and N9831 based on ClinicalTrials.gov last updated on 2021-04-29 (B-31) and 2020-08-14 (N9831). (DOCX)

S2 Table Operationalizations of eligibility criteria identified using codes in the emulation of a target trial comparing trastuzumab plus chemotherapy with chemotherapy, NKBC and seven further Swedish registers, 2008–2015. (DOCX)

S3 Table Overview of research aims, endpoints and operationalizations in the emulation of a target trial comparing trastuzumab plus chemotherapy with chemotherapy, NKBC and seven further Swedish registers, 2008–2015. (DOCX)

S4 Table Definitions and operationalizations of covariates used for all analyses in the emulation of a target trial comparing trastuzumab plus chemotherapy with chemotherapy, NKBC and seven further Swedish registers, 2008–2015. (DOCX)

S5 Table Final inverse probability of treatment weights truncated at the 99th percentile in the emulation of a target trial comparing trastuzumab plus chemotherapy with chemotherapy (main analysis), NKBC and seven further Swedish registers, 2008–2015. (DOCX)

S6 Table Survival, risk differences, and risk ratios at 5 years from baseline estimated in the observational emulation of a target trial comparing trastuzumab plus chemotherapy with chemotherapy, NKBC and seven further Swedish registers, 2008–2015 (complete case analysis based on 1,327 individuals). (DOCX)

S7 Table Survival, risk differences, and risk ratios at 5 years from baseline estimated in the observational emulation of a target trial comparing trastuzumab plus chemotherapy with chemotherapy, NKBC and seven further Swedish registers, 2008–2015 (sensitivity analysis restricted to individuals who initiated anthracyclines at baseline based on 940 individuals). (DOCX)

S8 Table Survival from breast cancer, risk differences, and risk ratios at 5 years from baseline estimated in the observational emulation of a target trial comparing trastuzumab plus chemotherapy with chemotherapy, NKBC and seven further Swedish registers, 2008–2015 (main analysis based on 1,578 individuals). (DOCX)

S1 Fig Inverse-probability weighted survival curve of overall survival to compare adjuvant chemotherapy + trastuzumab with chemotherapy from an observational emulation of a target trial, NKBC and seven further Swedish registers, 2008–2015 (main analysis based on 1,578 individuals). (EPS)

S2 Fig Inverse-probability weighted survival curves of the single recurrence components of the composite endpoint disease-free survival to compare adjuvant chemotherapy trastuzumab with chemotherapy from an observational emulation of a target trial, NKBC and seven further Swedish registers, 2008–2015 (main analysis based on 1,578 individuals). **(A)** Distant recurrence. **(B)** Local recurrence. **(C)** Contralateral breast cancer. **(D)** Other second primary cancer.
(EPS)

S3 Fig Inverse-probability weighted survival curve of disease-free survival to compare adjuvant chemotherapy + trastuzumab with chemotherapy from an observational emulation of a target trial, NKBC and seven further Swedish registers, 2008–2015 (complete case analysis based on 1,327 individuals). (EPS)

S4 Fig Inverse-probability weighted survival curve of overall survival to compare adjuvant chemotherapy + trastuzumab with chemotherapy from an observational emulation of a target trial, NKBC and seven further Swedish registers, 2008–2015 (complete case analysis based on 1,327 individuals). (EPS)

S5 Fig Inverse-probability weighted survival curves of the single recurrence components of the composite endpoint disease-free survival to compare adjuvant chemotherapy + trastuzumab with chemotherapy from an observational emulation of a target trial, NKBC and seven further Swedish registers, 2008–2015 (complete case analysis based on 1,327 individuals). **(A)** Distant recurrence. **(B)** Local recurrence. **(C)** Contralateral breast cancer. **(D)** Other second primary cancer. a. non-informative due to low event numbers in the chemotherapy arm.
(EPS)

S6 Fig Inverse-probability weighted survival curve of disease-free survival to compare adjuvant chemotherapy + trastuzumab with chemotherapy from an observational emulation of a target trial, NKBC and seven further Swedish registers, 2008–2015 (sensitivity analysis restricted to individuals who initiated anthracyclines at baseline based on 940 individuals). (EPS)

S7 Fig Inverse-probability weighted survival curve of overall survival to compare adjuvant chemotherapy + trastuzumab with chemotherapy from an observational emulation of a target trial, NKBC and seven further Swedish registers, 2008–2015 (sensitivity analysis restricted to individuals who initiated anthracyclines at baseline based on 940 individuals). (EPS)

S8 Fig Inverse-probability weighted survival curves of the single recurrence components of the composite endpoint disease-free survival to compare adjuvant chemotherapy + trastuzumab with chemotherapy from an observational emulation of a target trial, NKBC and seven further Swedish registers, 2008–2015 (sensitivity analysis restricted to individuals who initiated anthracyclines at baseline based on 940 individuals). **(A)** Distant recurrence. **(B)** Local recurrence. **(C)** Contralateral breast cancer. **(D)** Other second primary cancer. a. non-informative due to low event numbers in the chemotherapy arm.
(EPS)

S1 Appendix Coefficients from pooled logistic regression model for the denominator of the inverse probability of treatment weights (main analysis). (DOCX)

S1 Checklist STROBE Statement checklist of items that should be included in reports of observational studies. (PDF)

## Abbreviations

CI: confidence interval
HER2: human epidermal growth factor receptor 2
HR: hazard ratio
LVEF: left ventricular ejection fraction
RDs: risk differences
RRs: risk ratios
