# Supplementary material for: Trastuzumab in early curative breast cancer: A target trial emulation benchmarked against two randomized clinical trials
Source: PLoS Med. 2025 Jul 21;22(7):e1004661. doi: 10.1371/journal.pmed.1004661 (PMC12303387; doi:10.1371/journal.pmed.1004661)
Supplement: S1 Data — (DOCX) [file pmed.1004661.s001.docx]

S1 Data

In this target trial, the intention to treat effect is the effect of being assigned to trastuzumab plus chemotherapy versus chemotherapy on the composite of disease-free survival operationalized as the earliest of 1) local recurrence, 2) distant recurrence, 3) contralateral breast cancer, 4) other second primary cancer, or 5) death from any cause, and the effect on its single components. Its observational analog cannot be estimated if treatment strategies are indistinguishable at baseline, as a causal contrast based on the assignment to a treatment strategy at baseline would lead to a comparison of equivalent groups. The per-protocol effect is the effect of receiving and adhering to the assigned strategy of either trastuzumab plus chemotherapy or chemotherapy on the outcomes of interest. Estimating its observational analog when treatment strategies are indistinguishable (and therefore are the same for these individuals) at baseline requires modelling who adhered to their treatment strategy, which was assigned at this point in time. As described in the main body of the article, we applied the previously outlined approach of clone-censor weighting with a grace period.

In brief, this approach comprises that individuals contribute to both strategies as long as their data are compatible with both strategies (cloning). This avoids immortal time bias. Once they deviate from one of their assigned strategies, they no longer count towards this strategy (censoring). Censoring individuals at the time of treatment non-adherence is mathematically equivalent to assigning individuals a time-varying weight of 0 from that point in time. To account for potential selection bias introduced through censoring, we used inverse-probability of treatment (IP) weighting to create a pseudopopulation in which, given past treatment values, future treatment is independent from the measured baseline and time-varying covariates.

***IP weighting***

The stabilized IP weights as described below are estimated based on the original study population, prior to cloning, for each individual:

$$SW_{t}^{\bar{A}}=\prod_{n=0}^{t} \frac{f(\left. A_{n} \right|\overline{A}_{n-1}, \text{L}\text{0},\overline{Y}_{n}=0)}{f\left( \left. A_{n} \right|\overline{A}_{n-1},{\overline{L}_{n}, \overline{Y}}_{n}=0 \right)}$$

where:

$\overline{A}_{n-1}$: Values of prior treatment.

$\overline{Y}$_n_: History of outcome of interest at times n = t.

$\overline{L}$_n_: History of potentially confounding factors at times n = t.

We estimate the denominator of these time-varying IP weights by fitting the below described pooled logistic regression model for the weekly probability of receiving trastuzumab conditional on prior treatment and covariate history and within person-weeks with a positive probability of receiving trastuzumab based on the data. Informally, the denominator of these time-varying IP weights for each uncensored individual at time *t* is the probability that an individual remained uncensored up until this point in time given their past treatment values and baseline as well as time-varying covariates. All continuous covariates were modelled as linear and quadratic terms (see S4 Table). Time was modelled in weekly intervals as the function f(t) using linear and quadratic terms.

*logit*(P[𝐴_t_ = 1 | *cum*($\overline{A}$_t−1_) = 1, $\overline{L}$_t_, $\overline{Y}$_t_ = 0, K = 1]) = β_0, t_ + β^T^_1_ f(t) + β^T^_2_ 𝐿_0_ + β^T^_3_ 𝐿_t_

where:

K = 1: Times with a positive probability of receiving trastuzumab.

β_0, t_: Time-varying intercept, estimated as both a constant plus linear and quadratic term at time t.

The numerator of the stabilized IP weights is estimated analogously by fitting the below described pooled logistic regression model for the weekly probability of receiving trastuzumab conditional on prior treatment and baseline covariates. All continuous covariates including time in weeks were again modelled as linear and quadratic terms. The model was also restricted to person-weeks with a positive probability of receiving trastuzumab.

*logit*(P[𝐴_t_ = 1 | *cum*($\overline{A}$_t−1_) = 1, 𝐿_0_, $\overline{Y}$_t_ = 0, K = 1]) = β_0, t_ + β^T^_1_ f(t) + β^T^_2_ 𝐿_0_

Both, the model for the denominator and for the numerator, are thus fit only to person-weeks when trastuzumab initiation could occur in our dataset. At time points when no one could receive trastuzumab, all individuals contributed a weight of 1 (i.e., after week 62). The final weights were the cumulative product of time-specific weights across all time points. We truncate these weights at the 99^th^ percentile.

Finally, we fit the below pooled logistic regression model for the outcome of interest in which individuals are weighted by the truncated SW^A^, with a treatment strategy indicator, a function of time f(t) as linear and quadratic terms, and their interactions.

*logit*(P[Y_t+1_ = 1 | Y_t_ = 0, A) = β_0, t_ + β^T^_1_ f(t) + β_2_A + β^T^_3_ f(t)A

The survival, risks, risk differences, and risk ratios can be calculated. To calculate the 95% confidence intervals, we used nonparametric bootstrapping with 500 samples.
