# Supplementary material for: Trastuzumab in early curative breast cancer: A target trial emulation benchmarked against two randomized clinical trials
Source: PLoS Med. 2025 Jul 21;22(7):e1004661. doi: 10.1371/journal.pmed.1004661 (PMC12303387; doi:10.1371/journal.pmed.1004661)
Supplement: S1 Table — (DOCX) [file pmed.1004661.s002.docx]

**S1 Table. Summary of selected protocol components of the RCTs B-31 and N9831 based on ClinicalTrials.gov last updated on 2021-04-29 (B-31) and 2020-08-14 (N9831)**

| **Protocol component** | **Randomized trial B-31 – NCT00004067** | **Randomized trial N9831 – NCT00005970** |
| --- | --- | --- |
| Eligibility criteria^a^ | **Inclusion criteria**   - The patient must have a life expectancy of at least 10 years, excluding her diagnosis of breast cancer. (Comorbid conditions should be taken into consideration, but not the diagnosis of breast cancer.) - The interval between the last surgery for breast cancer treatment (lumpectomy, mastectomy, axillary dissection, or re-excision of lumpectomy margins) and randomization must be less than or equal to 84 days. - All of the following staging criteria must be met:   - Primary tumor must be T1-3 by clinical and pathologic evaluation.   - Ipsilateral nodes must be cN0-1 by clinical evaluation.   - Ipsilateral nodes must be pN1, pN2a, or pN3a by pathologic evaluation.   - M0 - Patients must have undergone either a total mastectomy and an axillary dissection or a lumpectomy and an axillary dissection. Sentinel node biopsy is permitted, but must be followed by an axillary dissection. - The tumor must be invasive adenocarcinoma on histologic examination. - The tumor must be determined to be HER2-positive prior to randomization. Assays performed using fluorescent in situ hybridization (FISH) require gene amplification to be eligible. Assays using immunohistochemistry (IHC) must be performed at an NSABP-approved reference laboratory and require a strongly positive staining score. - Patients must have an analysis of both estrogen and progesterone receptors performed on the primary tumor prior to randomization. "Marginal," "borderline," etc., results (i.e., those not definitely negative) will also be considered positive regardless of the methodology used. - At the time of randomization, the patient must have had the following: history and physical exam, EKG, and PA and lateral chest x-ray within the past 3 months; and a bilateral mammogram (or unilateral if patient has had a mastectomy) and a pelvic exam (for women who have a uterus and who will be taking tamoxifen) within the past year. - Within 3 months prior to entry, the patient must have a baseline left ventricular ejection fraction (LVEF) measured by MUGA scan equal to or greater than the lower limit of normal for the radiology facility. (If LVEF is > 75%, the investigator should consider having the LVEF determination reviewed prior to randomization. Following randomization, the LVEF determination may be reviewed up until the time of the post-AC MUGA. Please note that if a more accurate value is obtained from the review of the baseline MUGA, the corrected value must be submitted to the NSABP Biostatistical Center before the post-AC MUGA is performed.) - At the time of randomization:   - The postoperative absolute neutrophil count (ANC) must be ≥ 1500/mm3 (or <1500/mm3 if, in the opinion of the investigator, this represents an ethnic or racial variation of normal).   - Postoperative platelet count must be ≥ 100,000/mm3. Significant underlying hematologic disorders must be excluded when the platelet count is above the upper limit of normal for the lab. - There must be postoperative evidence of adequate hepatic function, i.e., total bilirubin must be ≤ ULN for the lab unless the patient has a chronic grade 1 bilirubin elevation (>ULN to ≤1.5 x ULN) due to Gilbert's disease or similar syndrome; and alkaline phosphatase must be <2.5 times the ULN for the lab; and the serum glutamic-oxaloacetic transaminase (SGOT [AST]) must be <1.5 times the ULN for the lab. - There must be postoperative evidence of adequate renal function (serum creatinine within or less than the institution's normal range). - Patients must have no clinical or radiologic evidence of metastatic disease. Suspicious findings must be confirmed as benign by radiologic evaluation or biopsy. A patient with skeletal pain is eligible for inclusion in the study if bone scan and/or roentgenological examination fails to disclose metastatic disease. - Patients with a history of non-breast malignancies are eligible if they have been disease-free for 5 or more years prior to randomization and are deemed by their physician to be at low risk for recurrence. Patients with the following cancers are eligible if diagnosed and treated within the past 5 years: carcinoma in situ of the cervix, melanoma in situ, and basal cell and squamous cell carcinoma of the skin. - Prior to randomization, the investigator must designate whether the patients who had a lumpectomy will receive local or locoregional radiation therapy. For patients who had a mastectomy, the investigator must designate whether or not the patient will receive radiation therapy. (Pre-randomization discussion and/or consultation with a radiation oncologist is encouraged.) Note: Irradiation of any internal mammary nodes is prohibited in this trial. - Special conditions for eligibility of lumpectomy patients: irradiation and surgery - Patients treated by lumpectomy and axillary node dissection to be followed by breast radiation therapy must meet all the eligibility criteria in addition to the following: Generally, lumpectomy should be reserved for tumors <5 cm. However, at the investigator's discretion, patients treated with lumpectomy for tumors ≥ 5 cm are eligible. The margins of the resected specimen must be histologically free of invasive tumor and DCIS as determined by the local pathologist. In patients in whom pathologic examination demonstrates tumor present at the line of resection, additional operative procedures may be performed to obtain clear margins. This is permissible even if axillary dissection has been performed. Patients in whom tumor is still present at the resected margin after re-excision(s) must undergo total mastectomy to be eligible. Whole breast irradiation is required. Irradiation of regional lymph nodes is optional, but partial breast irradiation and irradiation of any internal mammary nodes are prohibited in this trial. Intent to irradiate the axilla or other regional node groups must be declared by the investigator prior to randomization for stratification purposes. - Special conditions for eligibility of mastectomy patients: irradiation. The decision to use locoregional irradiation in patients who have undergone total mastectomy and axillary node dissection must be declared by the investigator prior to randomization for stratification purposes. Failure to adhere to the radiation therapy plan will be a protocol violation.   **Exclusion criteria**   - Bilateral malignancy or a mass or mammographic abnormality in the opposite breast suspicious for malignancy unless there is biopsy proof that the mass is not malignant. - Primary tumor staged as T4 for any reason. - Nodes staged as clinical N2 or N3 for any reason and nodes staged as pathologic pN2b, pN3b, or pN3c. - Prior history of breast cancer, including DCIS (patients with a history of lobular carcinoma in situ [LCIS] are eligible). - Treatment including radiation therapy, chemotherapy, biotherapy, and/or hormonal therapy administered for the currently diagnosed breast cancer prior to randomization. The only exception is hormonal therapy, which may have been given for up to a total of 28 days anytime after diagnosis and before randomization. In such a case, hormonal therapy must stop at or before randomization and be re-started if indicated following chemotherapy. - Prior anthracycline or taxane therapy for any malignancy. - Any sex hormonal therapy, e.g., birth control pills, ovarian hormonal replacement therapy, etc. (These patients are eligible only if this therapy is discontinued prior to randomization.) - Therapy with any hormonal agents such as raloxifene (Evista®), tamoxifen, or other selective estrogen receptor modulators (SERMs), either for osteoporosis or prevention. (Patients are eligible only if these medications are discontinued prior to randomization. These medications are not permitted while on the study except for the use of tamoxifen as described in the protocol) - Nonmalignant systemic disease (cardiovascular, renal, hepatic, etc.) that would preclude a patient from being subjected to any of the treatment options or would prevent prolonged follow-up. - Cardiac disease that would preclude the use of Adriamycin, Taxol or Herceptin. This includes: - Active cardiac disease:   - angina pectoris that requires the use of antianginal medication;   - cardiac arrhythmia requiring medication;   - severe conduction abnormality;   - clinically significant valvular disease;   - cardiomegaly on chest x-ray;   - ventricular hypertrophy on EKG; or   - patients with poorly controlled hypertension, i.e., diastolic greater than 100 mm/Hg. (Patients with hypertension who are well controlled on medication are eligible for entry.) - History of cardiac disease:   - myocardial infarction documented as a clinical diagnosis or by EKG or any other tests;   - documented congestive heart failure; or   - documented cardiomyopathy. - Psychiatric or addictive disorders that would preclude obtaining informed consent. - Pregnancy or lactation at the time of proposed randomization. This protocol excludes pregnant or lactating women based on the fetal toxicity of both tamoxifen and Taxol which are listed as Pregnancy Category D agents. Pregnant women who received tamoxifen have experienced fetal deaths, birth defects, spontaneous abortions, and vaginal bleeding. Women of reproductive potential must agree to use an effective barrier method of contraception. Hormonal birth control methods are not permitted. - Sensory/motor neuropathy ≥ grade 2, as defined by the NCI's Common Toxicity Criteria version 2.0. - Contraindications to corticosteroid use which, in the opinion of the investigator, would preclude participation in this study. - Concurrent treatment with other investigational agents. - Sensitivity to benzyl alcohol. - Special conditions for ineligibility of lumpectomy patients: irradiation and surgery. For patients treated by lumpectomy with axillary dissection, breast irradiation is required. Please see guidelines for radiation therapy in Appendix A. In addition, the following patients will also be ineligible:   - Patients with diffuse tumors (as demonstrated on mammography) that would not be considered surgically amenable to lumpectomy.   - Patients treated with lumpectomy in whom there is another clinically dominant mass or mammographically suspicious abnormality within the ipsilateral breast remnant. Such a mass must be biopsied and demonstrated to be histologically benign prior to randomization or, if malignant, must be surgically removed with clear margins.   - Patients in whom the margins of the resected specimen are involved with invasive tumor or ductal carcinoma in situ (DCIS). Additional surgical resections to obtain free margins are allowed. Patients in whom tumor is still present after the additional resection(s) must undergo mastectomy to be eligible. | **Inclusion Criteria**   - Required tumor parameters for node positive disease: NOTE: This study will continue to use the American Joint Committee on Cancer (AJCC) 5th edition for TNM classification and staging   - Operable, histologically confirmed adenocarcinoma of the female breast and positive lymph nodes     - Node positivity may be determined by either an axillary node dissection or a positive sentinel node finding by hematoxylin and eosin (H&E)       - NOTE: Positive nodes refers to H&E visible nodal metastases; immunohistochemistry (IHC) positive only cells in lymph nodes will not be considered positive nodes     - One or more positive lymph nodes whose tumors are T1-3, pN1-2, M0 are eligible     - cN2 disease is not eligible     - pN2 disease is eligible     - One positive lymph node by sentinel node biopsy or at least 6 axillary nodes must be examined on axillary node dissection with at least one positive lymph node     - Metaplastic carcinoma is eligible   - ER/PgR determination   - HER-2 positive (pre-entry requirement for registration)     - FISH must show gene amplification OR     - IHC assay must show a strong positive (3+) staining score       - NOTE: ductal carcinoma in situ (DCIS) components should not be counted in the determination of degree of IHC staining or FISH amplification - Required tumor parameters for high-risk node-negative disease; NOTE: This study will continue to use the AJCC 5th edition for TNM classification and staging   - Operable, histologically confirmed adenocarcinoma of the female breast and negative lymph nodes     - Node status may be determined by either axillary node dissection or sentinel node biopsy with H&E staining; to be considered node negative, either of the following must be true: 1) negative sentinel node biopsy or 2) no positive lymph nodes found among at least 6 axillary nodes examined on axillary node dissection     - NOTE: IHC positive only cells in lymph nodes will not be considered positive nodes     - Tumors > 2.0 cm (irrespective of hormonal receptor status) or > 1.0 cm if ER-negative and PR-negative disease   - ER/PgR determination   - HER-2 positive (pre-entry requirement for registration)     - FISH must show gene amplification OR     - IHC assay must show a strong positive (3+) staining score       - NOTE: DCIS components should not be counted in the determination of degree of IHC staining or FISH amplification - =< 84 days from mastectomy or =< 84 days from axillary dissection or sentinel node detection if the patient's most extensive breast surgery was a breast sparing procedure; (This timing is per a decision by the Breast Intergroup) - Surgical resection margins. All tumor should be removed by either a modified radical mastectomy or a segmental mastectomy with axillary node dissection   - Mastectomy: There will be no evidence of gross or microscopic tumor (invasive or DCIS) at the surgical resection margins noted in the final surgery or pathology reports; patients with close margins are eligible   - Segmental mastectomy (lumpectomy): Margins must be clear of invasive cancer and DCIS   - Axillary dissection or sentinel node dissection: There will be no gross residual adenopathy - TAM therapy   - May have received up to four weeks of TAM therapy, or any other hormonal agent, for this malignancy   - May have received TAM or raloxifene for purposes of chemoprevention (e.g., Breast Cancer Prevention Trial) or for other indications (including previous breast cancer if lobular carcinoma in situ [LCIS]) but must be discontinued before registration on this study   - May never have received TAM, raloxifene, or any other hormonal agent - Absolute neutrophil count (ANC) >= 1500/mm^3 - Platelets (PLT) >= 100,000/mm^3 - Total bilirubin =< 1.5 x upper normal limit (UNL) - Aspartate aminotransferase (AST) =< 2.0 x UNL - Left ventricular ejection fraction (LVEF) within institutional normal range; if LVEF is > 75%, the investigator should consider performing a second review of the multigated acquisition (MUGA)/echocardiogram or performing a repeat MUGA/echocardiogram prior to registration; such re-reviews or repeat MUGA/echocardiogram are not permitted after registration - Willingness to discontinue sex hormonal therapy, e.g., birth control pills, ovarian hormonal replacement therapy, etc., prior to registration and while on study - Willingness to discontinue any hormonal agent such as raloxifene (Evista) prior to registration and while on study - Non-breast malignancies that have not recurred within the last 5 years and are deemed to be at low risk for recurrence   **EXCEPTIONS:** These non-breast malignancies are eligible even if diagnosed =< 5 years prior to registration:   - Squamous or basal cell carcinoma of the skin that has been effectively treated - Carcinoma in situ of the cervix that has been treated by surgery only - Lobular carcinoma in situ (LCIS) of the ipsilateral or contralateral breast treated by surgery and/or tamoxifen only   - Patients undergoing breast conservation therapy (i.e., lumpectomy and axillary dissection) must have plans to receive radiation therapy to the breast +/- regional lymphatics following completion of the chemotherapy; for patients treated with mastectomy, the use of radiation therapy is required for 4 or more positive lymph nodes and must be started after completion of chemotherapy; the use of radiation therapy is at the discretion of the investigator for 0-3 positive lymph nodes but, if used, must be started after the completion of chemotherapy   - Prior to registration, the physician must designate if it is planned for the patient to receive radiation therapy (for adjuvant radiation therapy post-mastectomy or, less commonly, post-conservative therapy but not primary breast radiation as part of breast conserving treatment)   - Willing and able to sign an informed consent   - Gene amplified by FISH or strong positivity (3+) by HercepTest on central review; Note: The patient registers based on community HER-2 testing using FISH or IHC, AC chemotherapy is initiated; the tumor block or slides must be received =< 2 weeks from time of registration to the North Central Cancer Treatment Group (NCCTG) Operations Office for central HER-2 testing   **Exclusion Criteria**   - Any of the following:   - Pregnant women   - Nursing women   - Women of childbearing potential or their sexual partners who are unwilling to employ adequate contraception (condoms, diaphragm, intrauterine device [IUD], surgical sterilization, or abstinence, etc.); hormonal birth control methods are not permitted - Locally advanced tumors (classification T4) at diagnosis including tumors fixed to chest wall, peau d'orange, skin ulcerations/nodules, or clinical inflammatory changes (diffuse brawny cutaneous induration with an erysipeloid edge) - Prior history of breast cancer, except LCIS - Bilateral invasive carcinoma, either metachronous or synchronous (EXCEPTION: Patients diagnosed with unilateral invasive carcinoma and metachronous or synchronous DCIS of the contralateral breast treated with mastectomy are eligible) - Prior chemotherapy, radiation therapy, immunotherapy, or biotherapy for breast cancer - Active, unresolved infection - Active cardiac disease   - Any prior myocardial infarction   - History of documented congestive heart failure (CHF)   - Current use of digitalis or beta-blockers for CHF   - Any prior history of arrhythmia or cardiac valvular disease requiring medications or clinically significant   - Current use of medications for treatment of arrhythmias or angina pectoris   - Current uncontrolled hypertension (diastolic > 100 mmHg or systolic > 200 mmHg)   - Clinically significant pericardial effusion - Prior anthracycline or taxane therapy for any malignancy - Sensitivity to benzyl alcohol - Neurology/Neuropathy-Sensory >= grade 2 per the National Cancer Institute's (NCI's) Common Toxicity Criteria Version 2.0; EXCEPTION: Any chronic neurologic disorder will be looked at on a case-by-case basis by the study chair |
| Treatment strategies under investigation^b^ | (1) Initiation of trastuzumab concomitant to a taxane based chemotherapy after 12 weeks of receiving anthracycline-based combination chemotherapy  (2) No initiation of trastuzumab over follow-up while receiving the same chemotherapy regime as in the intervention arm | |
| Chemotherapies administered, doses, and durations of treatment^b^ | - Anthracycline-based combination chemotherapy: doxorubicin (60 mg/m^2^) and cyclophosphamide (600 mg/m^2^) every 21 days for four cycles - Taxane based chemotherapy: paclitaxel à 175 mg/m^2^ every 3 weeks for four cycles or at 80 mg/m^2^ for 12 weekly doses at the investigator’s discretion - Trastuzumab: loading dose of 4 mg/kg, followed by weekly doses of 2 mg/kg for 51 weeks | - Anthracycline-based combination chemotherapy: doxorubicin (60 mg/m^2^) and cyclophosphamide (600 mg/m^2^) every 21 days for four cycles - Taxane based chemotherapy: paclitaxel à 80 mg/m^2^ for 12 weekly doses - Trastuzumab: loading dose of 4 mg/kg, followed by weekly doses of 2 mg/kg for 51 weeks |
| Treatment assignment | Patients were randomized in a 1:1 ratio to one of the above described treatment arms. | Patients were randomized in a 1:1:1 ratio to one of the above described or the third below mentioned^b^ treatment arm. |
| Primary and secondary endpoints | Disease-free survival and overall survival  Disease-free survival operationalized as time from randomization until the earliest of 1) local or regional recurrences, 2) distant recurrences, 3) contralateral breast cancer, 4) other second primary cancer, or 5) death from any cause | |
| Planned systematic follow-up | 5 years | 15 years |
| Causal contrast | Intention-to-treat effect and per-protocol effect | |
| Statistical analysis | Intention-to-treat analysis and per-protocol analysis, primarily using:   - Kaplan-Meier method - Stratified log-rank test | |
| a. adopted from ClinicalTrials.gov  b. A third treatment arm in trial N9831 was to evaluate whether trastuzumab be added sequentially or concurrently to a taxane-based chemotherapy. As this question is outside the scope of this article, it is left out in this table to enhance readability. Furthermore, both trials had protocol specifications on radiation therapy and hormonal treatments to be administered, if indicated. | | |
