# Supplementary material for: Trastuzumab in early curative breast cancer: A target trial emulation benchmarked against two randomized clinical trials
Source: PLoS Med. 2025 Jul 21;22(7):e1004661. doi: 10.1371/journal.pmed.1004661 (PMC12303387; doi:10.1371/journal.pmed.1004661)
Supplement: S2 Table — (DOCX) [file pmed.1004661.s003.docx]

S2 Table. Operationalizations of eligibility criteria identified using codes in the emulation of a target trial comparing trastuzumab plus chemotherapy with chemotherapy, NKBC and seven further Swedish registers, 2008-2015

| **Exclusion criterion** | **Register used** | **Operationalization in codes** |
| --- | --- | --- |
| **Contraindications or trastuzumab related exclusion criteria** | | |
| ***Active cardiac diseases*** | | |
| Angina pectoris that requires the use of antianginal medication, cardiac arrhythmia requiring medication, or current use of digitalis or beta-blockers for congestive heart failure | Prescribed drug register | Any ATC code containing the following strings: C01A, C01B, C01D, C01E, C07, or C08 |
| Severe conduction abnormality, clinically significant valvular disease, clinically significant pericardial effusion, cardiomegaly on chest x-ray, ventricular hypertrophy on EKG, or LVEF outside the normal range | Outpatient and inpatient register | Any of the following ICD-10 codes captured as main diagnosis: I442, I443, I456, I457, I459, I4901, I4902, I493, I494, I495,  I359, I350, I351, I352, I080, I050, I052, I058, I340, I341, I070, I071, I078, I360, I361, I088, I089, I517, I515, I420, I426, I313, I501, I255, I420, I426, I429 |
| ***History of cardiac diseases*** | | |
| Myocardial infarction, congestive heart failure, or cardiomyopathy | Outpatient and inpatient register | Any of the following ICD-10 codes captured as main or secondary diagnosis: I502, I501, I5021, I5022, I5023, I5030, I5031, I5032, I5033, I5040, I5041, I5042, I5043, I509, I429, I420, I421, I422, I423, I424, I425, I426, I427, I428, or contained I21 or I22 |
| ***Non-cardiac contraindications*** | | |
| Hematopoietic disorders in history | Outpatient and inpatient register | Any of the following ICD-10 codes captured as main or secondary diagnosis: D610, Q774, D570, D56, D710, D800, E7522 |
|  | | |
| **History of non-breast malignancies and cancers eligible to be included** | | |
| History of non-breast malignancies | Cancer Register | Any malignancy except breast cancer (see below for ICD codes) |
| Carcinoma in situ of the cervix, melanoma in situ, and non-melanoma skin cancers staged as no more than TisN0M0, while in situ could also be determined based on SNOMED and FIGO records | Cancer Register | Any code containing one of the following strings for the respective ICD versions: ICD-10: D06, C44, D30; ICD-9: 233, 173, 172; ICD-7: 171, 191, 190, and any of the following SNOMED or FIGO codes: SNOMEDO10: 80702, 81402; SNOMED3: 80702, 81402; FIGO: 0 |
|  | | |
| No prior history of breast cancer | Cancer Register | Any code containing one of the following strings for the respective ICD versions: ICD-10: C50; ICD-9: 174; ICD-7: 170 |
| EKG: electrocardiogram; FIGO: International Federation of Gynecology and Obstetrics cancer staging system; LVEF: left ventricular ejection fraction; NKBC: Swedish National Quality Register for Breast Cancer; SNOMED: Systematized Nomenclature of Medicine Clinical Terms | | |
