# Supplementary material for: Trastuzumab in early curative breast cancer: A target trial emulation benchmarked against two randomized clinical trials
Source: PLoS Med. 2025 Jul 21;22(7):e1004661. doi: 10.1371/journal.pmed.1004661 (PMC12303387; doi:10.1371/journal.pmed.1004661)
Supplement: S3 Table — (DOCX) [file pmed.1004661.s004.docx]

S3 Table. Overview of research aims, endpoints and operationalizations in the emulation of a target trial comparing trastuzumab plus chemotherapy with chemotherapy, NKBC and seven further Swedish registers, 2008-2015

| **Aims** | **Endpoint** | **Register used** | **Operationalization** |
| --- | --- | --- | --- |
| Primary | DFS | As below | Composite of one of the below defined events, whichever occurred first. |
| Secondary | Death | 1. NKBC 2. Cause of death register | The NKBC was used to identify the date of death and data was compared to the cause of death register. For one individual with no month and day of death in the NKBC register, information could be complemented using the cause of death register. |
|  | Local recurrence | Inpatient register | Any of the following surgical procedure codes for operations due to local recurrence of breast cancer according to NOMESCO [1-4]: HAF 00, HAF 10, HAF 20, HAF 99 |
|  | Distant recurrence | Inpatient register and outpatient register | Any ICD-10 code of the following categories: C76 (Malignant neoplasm of other and ill-defined sites), C77 (Secondary and unspecified malignant neoplasm of lymph nodes), C78 (Secondary malignant neoplasm of respiratory and digestive organs), C79 (Secondary malignant neoplasm of other and unspecified sites) |
|  | Contralateral breast cancer | NKBC | Breast cancer diagnosis in the other side of the breast after baseline according to the NKBC. |
|  | Other second primary cancer | Cancer register | Any primary cancer except breast cancer (ICD-10 codes of category: C50). We additionally excluded few cases of secondary cancer (C76, C77, C78, and C79) entered in the cancer register. |
| DFS: disease-free survival; ICD: International Classification of Diseases; NKBC: Swedish National Quality Registry for Breast Cancer; NOMESCO: Nordic Medico-Statistical Committee | | | |

**References**

1. Nordic Medico-Statistical Committee (NOMESCO). NOMESCO Classification of Surgical Procedures (NCSP), version 1.13 2008 [updated Nov 20082023-11-18]. Available from: <https://rafhladan.is/bitstream/handle/10802/8074/NCSP_1_13.pdf?sequence=10>.

2. Nordic Medico-Statistical Committee (NOMESCO). NOMESCO Classification of Surgical Procedures (NCSP), version 1.14 2009 [updated Nov 20092023-11-18]. Available from: <https://norden.diva-portal.org/smash/get/diva2:970548/FULLTEXT01.pdf>.

3. Nordic Medico-Statistical Committee (NOMESCO). NOMESCO Classification of Surgical Procedures (NCSP), version 1.15 2010 [updated Nov 20102023-11-18]. Available from: <https://norden.diva-portal.org/smash/get/diva2:970547/FULLTEXT01.pdf>.

4. Nordic Medico-Statistical Committee (NOMESCO). NOMESCO Classification of Surgical Procedures (NCSP), version 1.16 2011 [updated Nov 20112023-11-18]. Available from: <https://norden.diva-portal.org/smash/get/diva2:968721/FULLTEXT01.pdf>.
