# Supplementary material for: Trastuzumab in early curative breast cancer: A target trial emulation benchmarked against two randomized clinical trials
Source: PLoS Med. 2025 Jul 21;22(7):e1004661. doi: 10.1371/journal.pmed.1004661 (PMC12303387; doi:10.1371/journal.pmed.1004661)
Supplement: S4 Table — (DOCX) [file pmed.1004661.s005.docx]

S4 Table. Definitions and operationalizations of covariates used for all analyses in the emulation of a target trial comparing trastuzumab plus chemotherapy with chemotherapy, NKBC and seven further Swedish registers, 2008-2015

| **Covariates** | **Register used** | **Full definitions, operationalizations, and categories (if applicable)** | **Form and proportion of NAs (if categorical)** |
| --- | --- | --- | --- |
| **Time-fixed** | | | |
| Age | NKBC | Age at diagnosis | Linear, quadratic |
| Marital status | LISA | Marital status in the year prior to primary diagnosis of breast cancer:  1 = not married  2 = married or registered partner  3 = divorced or divorced partner  4 = widow(er) or surviving partner  99 = NA | 5 categories  0.5% |
| Family income | LISA | Family income in the year prior to primary diagnosis of breast cancer:  1 = income below 25%-quantile  2 = income between 25%- and 75%-quantile  3 = income above 75%-quantile  99 = NA | 4 categories  0.5% |
| Menopausal status | NKBC | Menopausal status at diagnosis:  1 = premenopausal (<6 months after last menstruation)  2 = postmenopausal (≥ 6 months after last menstruation)  3 = more than 5 years postmenopausal  4 = uncertain (e.g., hysterectomy)  99 = NA | 5 categories  3.5% |
| Year of diagnosis | NKBC | 2008, 2009, 2010, 2011, 2012, 2013, 2014, or 2015 | 8 categories |
| Size of tumor at diagnosis | NKBC | Size of tumor at diagnosis according to TNM classification:  10 = T1, tumor ≤ 2.0 cm  20 = T2, tumor > 2.0 to 5.0 cm  30 = T3, tumor ≥ 5.1 cm  99 = NA or remote metastases cannot be assessed | 4 categories  0.2% |
| Lymph node involvement | NKBC | Lymph node involvement at diagnosis according to TNM classification:  0 = N0, no regional lymph node metastases  10 = N1, free lymph node metastasis(es) in axillary ipsilateral  99 = NA | 3 categories  0% |
| ER status | NKBC | ER status at diagnosis:  1 = positive  2 = negative  99 = NA | 3 categories  0.3% |
| PR status | NKBC | PR status at diagnosis:  1 = positive  2 = negative  99 = NA | 3 categories  0.5% |
| **Covariates** | **Register used** | **Full definitions, operationalizations, and categories (if applicable)** | **Form** |
| **Time-fixed** | | | |
| Size of the largest tumor at surgery | NKBC | 1 = ≤ 2.0 cm  2 = 2.1-4.0 cm  3 = ≥ 4.1 cm  99 = NA | 4 categories  1.3% |
| Histological grade | NKBC | Histological grade at surgery:  1 = grade 1  2 = grade 2  3 = grade 3  99 = NA | 4 categories  1.5% |
| Number of tumors in breast | NKBC | Number of tumors in breast at surgery | Linear, quadratic |
| Number of visits to healthcare professional within 5 years prior to baseline | Outpatient and inpatient register | Number of visits to healthcare professional within 5 years prior to baseline | Linear, quadratic |
| Week from baseline | - | - | Linear, quadratic |
| **Time-varying** | | | |
| Drug intake against cardiac disease as per eligibility criteria | Prescribed drug register | Angina pectoris that requires the use of antianginal medication, cardiac arrhythmia requiring medication, or current use of digitalis or beta-blockers for congestive heart failure (see eTable 2 for ICD‑10 codes related to these conditions) | Binary indicator |
| Mild to moderate cardiac disease as per eligibility criteria | Outpatient and inpatient register | Severe conduction abnormality, clinically significant valvular disease, clinically significant pericardial effusion, cardiomegaly on chest x-ray, ventricular hypertrophy on EKG, or LVEF outside the normal range (see eTable 2 for ICD‑10 codes related to these conditions) | Binary indicator |
| Severe cardiac disease as per eligibility criteria | Outpatient and inpatient register | Myocardial infarction, congestive heart failure, or cardiomyopathy (see eTable 2 for ICD‑10 codes related to these conditions; for defining the covariate, these were identified if captured as main diagnosis) | Binary indicator |
| Liver function disorder | Outpatient and inpatient register | Any ICD-10 code of the following category captured as main diagnosis: K7 (Diseases of liver) | Binary indicator |
| Renal function disorder | Outpatient and inpatient register | Any ICD-10 code of the following categories captured as main diagnosis: N17 (Acute kidney failure), N18 (Chronic kidney disease), N19 (Unspecified kidney failure), N25 (Disorders resulting from impaired renal tubular function), N28 (Other disorders of kidney and ureter, not elsewhere classified), or N29 (Other disorders of kidney and ureter in diseases classified elsewhere) | Binary indicator |
| **Covariates** | **Register used** | **Full definitions, operationalizations, and categories (if applicable)** | **Form** |
| **Time-varying** | | | |
| Dyspnea | Outpatient and inpatient register | Any ICD-10 code of the following category captured as main diagnosis: R06 (Abnormalities of breathing) | Binary indicator |
| Hematological events | Outpatient and inpatient register | Any of the following ICD-10 codes captured as main or secondary diagnosis: D701 (Drug-induced agranulocytosis and neutropenia), D707 (Neutropenia, unspecified), D728 (Other specified disorders of white blood cells), D729 (Disorder of white blood cells, unspecified), D630 (Anaemia in neoplastic disease), D6481 (Anemia due to antineoplastic chemotherapy), D695 (Secondary thrombocytopenia), D696 (Thrombocytopenia, unspecified), or R58 (Haemorrhage, not elsewhere classified) | Binary indicator |
| Severe infections | Outpatient and inpatient register | Any of the following ICD-10 codes or categories captured as main diagnosis: T802 (Infection sepsis following infusion, transfusion or therapeutic injection), A40 (Streptococcal sepsis), A41 (Other sepsis), J100 (Influenza with pneumonia, seasonal influenza virus identified), J110 (Influenza with pneumonia, virus not identified), J13 (Pneumonia due to Streptococcus pneumonia), J14 (Pneumonia due to Haemophilus influenza), J15 (Bacterial pneumonia, not elsewhere classified), J16 (Pneumonia due to other infectious organisms, not elsewhere classified), J17 (Pneumonia in diseases classified elsewhere), or J18 (Pneumonia, organism unspecified) | Binary indicator |
| Neurologic disorders | Outpatient and inpatient register | Any of the following ICD-10 codes captured as main diagnosis: G620 (Drug-induced polyneuropathy), G622 (Polyneuropathy due to other toxic agents), G629 (Polyneuropathy, unspecified), or G631 (Polyneuropathy in neoplastic disease) | Binary indicator |
| Gastrointestinal disorders | Outpatient and inpatient register | Any of the following ICD-10 codes or categories captured as main diagnosis: R11 (Nausea and vomiting), K521 (Toxic gastroenteritis and colitis), or K529 (Noninfective gastroenteritis and colitis, unspecified) | Binary indicator |
| ER: estrogen receptor; ICD: International Classification of Diseases; LISA: Longitudinal Integration Database for Health Insurance and Labor Market Studies; NKBC: Swedish National Quality Registry for Breast Cancer; PR: progesterone receptor | | | |
