# Supplementary material for: Trastuzumab in early curative breast cancer: A target trial emulation benchmarked against two randomized clinical trials
Source: PLoS Med. 2025 Jul 21;22(7):e1004661. doi: 10.1371/journal.pmed.1004661 (PMC12303387; doi:10.1371/journal.pmed.1004661)
Supplement: S5 Table — (DOCX) [file pmed.1004661.s006.docx]

S6 Table. Survival, risk differences, and risk ratios at 5 years from baseline estimated in the observational emulation of a target trial comparing trastuzumab plus chemotherapy with chemotherapy, NKBC and seven further Swedish registers, 2008-2015 (complete case analysis based on 1327 individuals)

|  | **Trastuzumab + chemotherapy** | | **Chemotherapy** | |  |  |
| --- | --- | --- | --- | --- | --- | --- |
| **Endpoint** | **Number of events (unique^a^)** | **Survival, %  (95% CI)** | **Number of events (unique^a^)** | **Survival, %  (95% CI)** | **Risk Difference, %  (95% CI)** | **Risk Ratio (95% CI)** |
| Disease-free survival | 198 (132) | 74.8 (70.8, 78.6) | 88 (22) | 63.2 (51.8, 73.3) | -11.6 (-23.1, -0.5) | 0.69 (0.50, 0.98) |
| Overall survival | 57 (52) | 90.9 (88.3, 93.4) | 21 (16) | 74.3 (63.9, 85.1) | -16.6 (-27.7, -5.9) | 0.35 (0.22, 0.61) |
| Local recurrence | 0 | –^b^ | 0 | –^b^ | –^b^ | –^b^ |
| Distant recurrence | 161 (101) | 80.5 (77.1, 83.7) | 76 (16) | 72.1 (62.2, 82.1) | -8.5 (-18.7, 2.6) | 0.70 (0.50, 1.15) |
| Contralateral breast cancer | 10 (9) | 97.8 (96.1, 99.3) | 2 (1) | 98.9 (95.9, 100.0) | 1.0 (-2.3, 3.4) | 1.94 (–^c^) |
| Other second primary cancer | 13 (13) | 98.1 (97.0, 99.2) | 1 (1) | 99.1 (97.3, 100.0) | 0.9 (-1.3, 2.9) | 2.04 (–^c^) |
| a. non-unique events resulting from months in which individuals contributed to both strategies and therefore counted towards both strategies  b. no events observed in any arm  c. non-informative due to low event numbers in the chemotherapy arm | | | | | | |
