# Supplementary material for: Trastuzumab in early curative breast cancer: A target trial emulation benchmarked against two randomized clinical trials
Source: PLoS Med. 2025 Jul 21;22(7):e1004661. doi: 10.1371/journal.pmed.1004661 (PMC12303387; doi:10.1371/journal.pmed.1004661)
Supplement: S7 Table — (DOCX) [file pmed.1004661.s008.docx]

S7 Table. Survival, risk differences, and risk ratios at 5 years from baseline estimated in the observational emulation of a target trial comparing trastuzumab plus chemotherapy with chemotherapy, NKBC and seven further Swedish registers, 2008-2015 (sensitivity analysis restricted to individuals who initiated anthracyclines at baseline based on 940 individuals)

|  | **Trastuzumab + chemotherapy** | | **Chemotherapy** | |  |  |
| --- | --- | --- | --- | --- | --- | --- |
| **Endpoint** | **Number of events (unique^a^)** | **Survival, %  (95% CI)** | **Number of events (unique^a^)** | **Survival, %  (95% CI)** | **Risk Difference, %  (95% CI)** | **Risk Ratio (95% CI)** |
| Disease-free survival | 147 (85) | 72.1 (66.6, 77.5) | 82 (20) | 64.7 (51.4, 77.6) | -7.4 (-21.3, 5.6) | 0.79 (0.55, 1.24) |
| Overall survival | 35 (31) | 91.1 (88.0, 94.0) | 18 (14) | 76.1 (63.0, 87.9) | -15.0 (-28.2, -3.0) | 0.37 (0.20, 0.76) |
| Local recurrence | 0 | –^b^ | 0 | –^b^ | –^b^ | –^b^ |
| Distant recurrence | 121 (64) | 78.6 (73.8, 82.9) | 72 (15) | 71.7 (59.2, 83.3) | -6.9 (-20.4, 4.7) | 0.76 (0.48, 1.26) |
| Contralateral breast cancer | 5 (4) | 98.3 (96.0, 99.7) | 2 (1) | 98.8 (95.7, 100.0) | 0.5 (-3.0, 3.2) | 1.41 (–^c^) |
| Other second primary cancer | 11 (11) | 97.5 (95.5, 99.0) | 1 (1) | 99.0 (97.0, 100.0) | 1.5 (-1.1, 4.2) | 2.59 (–^c^) |
| a. non-unique events resulting from months in which individuals contributed to both strategies and therefore counted towards both strategies  b. no events observed in any arm  c. non-informative due to low event numbers in the chemotherapy arm | | | | | | |
