# Supplementary material for: Trastuzumab in early curative breast cancer: A target trial emulation benchmarked against two randomized clinical trials
Source: PLoS Med. 2025 Jul 21;22(7):e1004661. doi: 10.1371/journal.pmed.1004661 (PMC12303387; doi:10.1371/journal.pmed.1004661)
Supplement: S8 Table — (DOCX) [file pmed.1004661.s009.docx]

S8 Table. Survival from breast cancer, risk differences, and risk ratios at 5 years from baseline estimated in the observational emulation of a target trial comparing trastuzumab plus chemotherapy with chemotherapy, NKBC and seven further Swedish registers, 2008-2015 (main analysis based on 1578 individuals)

|  | **Trastuzumab + chemotherapy** | | **Chemotherapy** | |  |  |
| --- | --- | --- | --- | --- | --- | --- |
| **Endpoint** | **Number of events (unique^a^)** | **Survival, %  (95% CI)** | **Number of events (unique^a^)** | **Survival, %  (95% CI)** | **Risk Difference, %  (95% CI)** | **Risk Ratio (95% CI)** |
| Survival from breast cancer | 42 (39) | 92.9 (90.5, 95.2) | 53 (50) | 79.3 (73.7, 84.2) | -13.6 (-19.3, -8.0) | 0.34 (0.22, 0.51) |
| a. non-unique events resulting from months in which individuals contributed to both strategies and therefore counted towards both strategies | | | | | | |
