# Supplementary material for: Trastuzumab in early curative breast cancer: A target trial emulation benchmarked against two randomized clinical trials
Source: PLoS Med. 2025 Jul 21;22(7):e1004661. doi: 10.1371/journal.pmed.1004661 (PMC12303387; doi:10.1371/journal.pmed.1004661)
Supplement: S1 Appendix — (DOCX) [file pmed.1004661.s018.docx]

S1 Appendix. Coefficients from pooled logistic regression model for the denominator of the inverse probability of treatment weights (main analysis).

| **Term** | **Estimate** | **Standard error** |
| --- | --- | --- |
| (Intercept) | -7,781862 | 0,708448 |
| age | 0,144067 | 0,022098 |
| I(age^2) | -0,001543 | 0,000185 |
| time | 0,090529 | 0,011287 |
| timesq | -0,00407 | 0,000381 |
| mari_stat_cat2 | -0,016681 | 0,086113 |
| mari_stat_cat3 | -0,025638 | 0,102498 |
| mari_stat_cat4 | -0,074662 | 0,150649 |
| mari_stat_cat99 | -0,116101 | 0,43743 |
| income_fam_cat2 | 0,086537 | 0,089085 |
| income_fam_cat3 | 0,234587 | 0,109116 |
| income_fam_cat99 | - | - |
| as.factor(menopausal_status)2 | 0,319733 | 0,11275 |
| as.factor(menopausal_status)4 | 0,096296 | 0,136189 |
| as.factor(menopausal_status)99 | 0,066749 | 0,173404 |
| as.factor(diag_dt_year)2009 | 0,410035 | 0,185125 |
| as.factor(diag_dt_year)2010 | 0,710106 | 0,173355 |
| as.factor(diag_dt_year)2011 | 0,611333 | 0,164748 |
| as.factor(diag_dt_year)2012 | 1,098048 | 0,166708 |
| as.factor(diag_dt_year)2013 | 1,239115 | 0,165222 |
| as.factor(diag_dt_year)2014 | 1,105262 | 0,165057 |
| as.factor(diag_dt_year)2015 | 1,196503 | 0,191784 |
| as.factor(diag_tclass)20 | -0,022107 | 0,081717 |
| as.factor(diag_tclass)30 | -0,076473 | 0,151962 |
| as.factor(diag_tclass)99 | 0,881043 | 0,628865 |
| as.factor(diag_nclass)10 | -0,013832 | 0,074617 |
| as.factor(diag_er)2 | 0,06125 | 0,091367 |
| as.factor(diag_er)99 | -0,292231 | 1,286789 |
| as.factor(diag_pr)2 | -0,028563 | 0,089641 |
| as.factor(diag_pr)99 | -0,692515 | 0,740759 |
| as.factor(op_grade)2 | 0,308894 | 0,317494 |
| as.factor(op_grade)3 | 0,617431 | 0,313252 |
| as.factor(op_grade)99 | 0,111758 | 0,419528 |
| op_numtumours | 0,046998 | 0,044308 |
| I(op_numtumours^2) | -0,000433 | 0,000442 |
| as.factor(op_sizetumour_cat)2 | 0,133231 | 0,074573 |
| as.factor(op_sizetumour_cat)3 | 0,218018 | 0,113449 |
| as.factor(op_sizetumour_cat)99 | -1,150374 | 0,411153 |
| conf_cardio_drug | -12,388327 | 605,76653 |
| conf_cardio_drug_lag1 | -12,369474 | 603,93957 |
| conf_cardio_mild | -12,54427 | 502,600918 |
| conf_cardio_mild_lag1 | -12,556819 | 502,172002 |
| conf_cardio_severe | -11,866374 | 322,053721 |
| conf_cardio_severe_lag1 | -11,858305 | 320,915092 |
| conf_liver_fu | - | - |
| conf_liver_fu_lag1 | - | - |
| conf_renal_fu | -12,423196 | 921,155698 |
| conf_renal_fu_lag1 | -12,396122 | 917,798843 |
| conf_dyspnea_fu | -12,984309 | 397,975463 |
| conf_dyspnea_fu_lag1 | -13,049858 | 414,822135 |
| conf_hemato_fu | 1,206283 | 0,783555 |
| conf_hemato_fu_lag1 | 0,59188 | 1,063139 |
| conf_infect_fu | -12,86653 | 288,083168 |
| conf_infect_fu_lag1 | 0,365231 | 0,757925 |
| conf_neuro_fu | -12,164903 | 1455,397543 |
| conf_neuro_fu_lag1 | -12,178094 | 1455,397543 |
| conf_gastro_fu | -0,22725 | 1,045115 |
| conf_gastro_fu_lag1 | -13,017983 | 373,90384 |
| conf_visits_5yr_bl | 0,004255 | 0,007234 |
| I(conf_visits_5yr_bl^2) | -0,000075 | 0,000119 |
