# Supplementary material for: Trastuzumab in early curative breast cancer: A target trial emulation benchmarked against two randomized clinical trials
Source: PLoS Med. 2025 Jul 21;22(7):e1004661. doi: 10.1371/journal.pmed.1004661 (PMC12303387; doi:10.1371/journal.pmed.1004661)
Supplement: S1 Checklist — (PDF) [file pmed.1004661.s019.pdf]

S1 Checklist

STROBE Statement—checklist of items that should be included in reports of observational studies.

	Item No	Recommendation	Reported in
**Title and abstract**	1	(*a*) Indicate the study’s design with a commonly used term in the title or the abstract	▪ Title and Abstract
		(*b*) Provide in the abstract an informative and balanced summary of what was done and what was found	▪ Abstract
**Introduction**			
Background/rationale	2	Explain the scientific background and rationale for the investigation being reported	▪ Introduction, 1st and 2nd paragraph
Objectives	3	State specific objectives, including any prespecified hypotheses	▪ Introduction, 3rd paragraph
**Methods**			
Study design	4	Present key elements of study design early in the paper	▪ Table 1<br>▪ Sections “Index randomized trials: B-31 and N9831”, 1st paragraph<br>▪ Section “The observational analysis”, “Target trial protocol”, particularly 1st and 2nd paragraph
Setting	5	Describe the setting, locations, and relevant dates, including periods of recruitment, exposure, follow-up, and data collection	▪ Table 1<br>▪ Section “The observational emulation of the target trial”, 1st paragraph
Participants	6	(*a*) *Cohort study*—Give the eligibility criteria, and the sources and methods of selection of participants. Describe methods of follow-up<br>*Case-control study*—Give the eligibility criteria, and the sources and methods of case ascertainment and control selection. Give the rationale for the choice of cases and controls<br>*Cross-sectional study*—Give the eligibility criteria, and the sources and methods of selection of participants	▪ Table 1 and S2 Table<br>▪ Section “The observational emulation of the target trial”, 2nd paragraph<br>▪ Follow-up is specifically addressed in Table 1 and in the Section “The observational analysis”, “Target trial protocol”, 2nd paragraph

Participants (continued)		(*b*) *Cohort study*—For matched studies, give matching criteria and number of exposed and unexposed<br>*Case-control study*—For matched studies, give matching criteria and the number of controls per case	▪ NA (no matching study)
Variables	7	Clearly define all outcomes, exposures, predictors, potential confounders, and effect modifiers. Give diagnostic criteria, if applicable	▪ Section "The observational emulation of the target trial", 2nd and 3rd paragraph<br>▪ S3 Table and S4 Table
Data sources/ measurement	8*	For each variable of interest, give sources of data and details of methods of assessment (measurement). Describe comparability of assessment methods if there is more than one group	▪ S2 Table, S3 Table, and S4 Table<br>▪ Section "The observational emulation of the target trial", 2nd paragraph
Bias	9	Describe any efforts to address potential sources of bias	▪ Introduction, 1st paragraph and throughout the manuscript (as benchmarking is used to increase confidence in observational data)<br>▪ Section "The observational emulation of the target trial", 3rd paragraph
Study size	10	Explain how the study size was arrived at	▪ Section "Results", 1st paragraph and Fig 1
Quantitative variables	11	Explain how quantitative variables were handled in the analyses. If applicable, describe which groupings were chosen and why	▪ S1 Data and S4 Table
Statistical methods	12	(*a*) Describe all statistical methods, including those used to control for confounding	▪ Section "The observational analysis", "Target trial protocol", 3rd paragraph<br>▪ Section "The observational emulation of the target trial", 3rd paragraph
		(*b*) Describe any methods used to examine subgroups and interactions	▪ NA
		(*c*) Explain how missing data were addressed	▪ Section "The observational emulation of the target trial", 2nd paragraph
		(*d*) *Cohort study*—If applicable, explain how loss to follow-up was addressed<br>*Case-control study*—If applicable, explain how matching of cases and controls was addressed<br>*Cross-sectional study*—If applicable, describe analytical methods taking account of sampling strategy	▪ Table 1, row "Benchmark statistical analysis", column "Target trial emulation using NKBC and seven other national Swedish registries"
		(*e*) Describe any sensitivity analyses	▪ Section "Sensitivity analyses"

**Results**			
Participants	13*	(a) Report numbers of individuals at each stage of study—eg numbers potentially eligible, examined for eligibility, confirmed eligible, included in the study, completing follow-up, and analysed	▪ Section "Results", 1st paragraph and Fig 1
		(b) Give reasons for non-participation at each stage	▪ Fig 1
		(c) Consider use of a flow diagram	▪ Fig 1
Descriptive data	14*	(a) Give characteristics of study participants (eg demographic, clinical, social) and information on exposures and potential confounders	▪ Section "Results", 1st paragraph<br>▪ Table 2
		(b) Indicate number of participants with missing data for each variable of interest	▪ S4 Table
		(c) *Cohort study*—Summarise follow-up time (eg, average and total amount)	▪ Section "The observational emulation of the target trial", 2nd paragraph
Outcome data	15*	*Cohort study*—Report numbers of outcome events or summary measures over time	▪ Section "Results", 2nd and 3rd paragraph<br>▪ Table 3 and all other results Tables (S6 Table, S7 Table, S8 Table)
		Case-control study—Report numbers in each exposure category, or summary measures of exposure	
		Cross-sectional study—Report numbers of outcome events or summary measures	
Main results	16	(*a*) Give unadjusted estimates and, if applicable, confounder-adjusted estimates and their precision (eg, 95% confidence interval). Make clear which confounders were adjusted for and why they were included	▪ Section "Results", 2nd and 3rd paragraph<br>▪ Table 3 and all other results tables (S6 Table, S7 Table, S8 Table)<br>▪ Factors adjusted for: section "The observational emulation of the target trial", 3rd paragraph
		(*b*) Report category boundaries when continuous variables were categorized	▪ S4 Table
		(*c*) If relevant, consider translating estimates of relative risk into absolute risk for a meaningful time period	▪ Survival proportions (100-risks) reported in all results tables (Table 3, S6 Table, S7 Table, S8 Table)
Other analyses	17	Report other analyses done—eg analyses of subgroups and interactions, and sensitivity analyses	▪ Section "Results", 3rd paragraph
**Discussion**			
Key results	18	Summarise key results with reference to study objectives	▪ Discussion, 1st paragraph

Limitations	19	Discuss limitations of the study, taking into account sources of potential bias or imprecision. Discuss both direction and magnitude of any potential bias	▪ Discussion, 3rd, 4th, 5th, 6th, 7th, and 8th paragraph
Interpretation	20	Give a cautious overall interpretation of results considering objectives, limitations, multiplicity of analyses, results from similar studies, and other relevant evidence	▪ Section "Conclusion"
Generalisability	21	Discuss the generalisability (external validity) of the study results	▪ Section "Implications and further research"<br>▪ Section "Conclusion"
**Other information**			
Funding	22	Give the source of funding and the role of the funders for the present study and, if applicable, for the original study on which the present article is based	▪ Section "Financial Disclosure"

*Give information separately for cases and controls in case-control studies and, if applicable, for exposed and unexposed groups in cohort and cross-sectional studies.

Note: An Explanation and Elaboration article discusses each checklist item and gives methodological background and published examples of transparent reporting. The STROBE checklist is best used in conjunction with this article (freely available on the Web sites of PLoS Medicine at http://www.plosmedicine.org/, Annals of Internal Medicine at http://www.annals.org/, and Epidemiology at http://www.epidem.com/). Information on the STROBE Initiative is available at www.strobe-statement.org.
